# Hidden Preeclampsia Leading to Placental Abruption and Disseminated Intravascular Coagulation

**DOI:** 10.7759/cureus.83469

**Published:** 2025-05-04

**Authors:** Nivedita Biju, Corinne Backer

**Affiliations:** 1 Obstetrics and Gynecology, A.T. Still University, Saint Louis, USA; 2 Obstetrics and Gynecology, Mercy Health, Saint Louis, USA

**Keywords:** atypical preeclampsia, disseminated intravascular coagulation (dic), eclampsia management, emergency obstetrics, placental abruption, postpartum hemorrhage (pph), preeclampsia

## Abstract

Placental abruption is a life-threatening obstetric emergency, often associated with hypertensive disorders such as preeclampsia. This study describes a 28-year-old multiparous woman at 36 weeks and four days of gestation who presented with contractions without any overt signs of preeclampsia. Despite an initial blood pressure of 130/85 mmHg, fetal bradycardia and uterine tachysystole prompted an emergency cesarean section. Intraoperative findings confirmed placental abruption, and postoperative laboratory results revealed significant proteinuria, confirming undiagnosed preeclampsia. Postpartum complications included severe hemorrhage exceeding 2000 mL, progressing to disseminated intravascular coagulation (DIC), which required aggressive resuscitation, a massive transfusion protocol, and intensive care management. This case emphasized the challenge of diagnosing "hidden" preeclampsia, the variability of placental abruption presentations, and the importance of early recognition and intervention. Clinicians should maintain a high index of suspicion for atypical presentations of preeclampsia to mitigate adverse maternal and fetal outcomes.

## Introduction

Placental abruption is an extremely serious complication in pregnancy and is characterized as a premature separation of the placenta from the uterine wall prior to delivery [1]. Placental abruption can be detrimental as it deprives the baby of oxygen and nutrients and causes heavy bleeding in the mother. Abruption is most likely to occur in the third trimester and can present with vaginal bleeding, abdominal pain, back pain, uterine tenderness and/or rigidity, and highly frequent uterine contractions [1]. Unfortunately, the abdominal and back pain often begin suddenly, and the amount of vaginal bleeding can vary greatly. There is also no way to tell how much of the placenta has separated from the uterus based on presentation, making the process highly unpredictable, and it can delay diagnosis and intervention [2]. For example, it is possible for blood to be trapped inside the uterus during a complete placental abruption and it may present with no vaginal bleeding [2]. Some cases may also present chronically, which may present with light, intermittent bleeding, slow fetal growth, and/or low amniotic fluid [1].

Placental abruption is relatively rare and occurs in 0.4-1% of pregnancies; however, it is a leading cause of maternal and perinatal morbidity and mortality [3]. Risk factors for placental abruption include hypertension, trauma, smoking, and a prior history of abruption [4].

A hypertensive disorder in pregnancy is preeclampsia, which affects 5-7% of pregnancies worldwide, and is a well-known risk factor for placental abruption [5]. It is classically defined as hypertension greater than or equal to 140/90 and proteinuria after 20 weeks of gestation [6]. Sometimes it may present with end-organ dysfunction without overt proteinuria [6]. In some cases, preeclampsia may be hidden as it presents with minimal or atypical symptoms, which leads to delayed recognition and an increased risk of severe complications such as placental abruption, eclampsia (preeclampsia with seizures), or preeclampsia with hemolysis, elevated liver enzymes, and low platelet counts (HELLP) syndrome [7].

This study details a rare presentation of placental abruption in a patient with undiagnosed, or “hidden,” preeclampsia. It highlights the diagnostic challenges, clinical management, and potential implications for obstetric care. By discussing this case, we aimed to emphasize the need for increased vigilance in identifying atypical presentations of preeclampsia to improve maternal and fetal outcomes.

## Case presentation

History of present illness

A 28-year-old G6P2315 female at 36 weeks and four days of gestation presented to the labor and delivery triage department with contractions. At the time, she denied loss of fluids or vaginal bleeding. The patient has a history of preeclampsia and fetal growth restriction, and all previous births were spontaneous vaginal births. Due to her history of preeclampsia, the patient was taking 81 mg of aspirin daily as a preventative medication after 12 weeks of gestation, which is a guideline recommended by the American College of Obstetrics and Gynecologists as well as the United States Preventive Services Taskforce [8].

Fetal heart tones were found to be in the 80s beats per minute (bpm) upon arrival, and resuscitation efforts were initiated. After administering one dose of terbutaline subcutaneously, the uterus had relaxed only slightly. Terbutaline, a beta-2 agonist used to reduce uterine contractions, was administered in an attempt to improve fetal heart rate through uteroplacental blood flow optimization [9]. The fetal heart rate improved to the 100s for less than 30 seconds but then remained between 80 and 100 beats per minute. Various position changes were attempted, but there seemed to be no changes in fetal heart tones. Artificial rupture of membranes was performed, revealing clear amniotic fluid. A fetal scalp electrode and an intrauterine pressure catheter were placed without difficulty. Despite all these interventions, the fetal heart rate did not improve. Therefore, the decision was made to proceed with a cesarean section for an emergency delivery of the baby.

Review of systems

The patient denied experiencing any fevers or chills. She reported no chest pain and no shortness of breath or cough. She denied nausea, vomiting, diarrhea, or constipation. Genitourinary symptoms, including dysuria or vaginal bleeding, are also absent. Neurologically, she reported no headaches or visual changes. Psychiatrically, she denied any current symptoms of depression or anxiety.

Physical findings

On admission, the patient’s blood pressure was 130/85 mmHg. She was awake, alert, and oriented, in no acute distress, but experiencing painful contractions. Abdominal examination revealed a gravid uterus that was firm but relaxed between contractions. Contractions were frequent and strong. Extremities were non-tender, with no edema noted.

On pelvic examination, the cervix was 4 cm dilated. Fetal heart tones were in the 80s upon arrival, and tocometry showed contractions occurring every minute. A limited bedside ultrasound was performed to assess fetal heart tones, confirming a fetal heart rate in the 80s.

Initial differential diagnosis and initial plan

As mentioned, the consistently low fetal heart tones, regardless of resuscitation efforts, prompted emergency cesarean section. A normal fetal heart rate ranges from 110 to 160 beats per minute, and fetal bradycardia is defined as a heart rate of less than 110 beats per minute lasting for at least 2 minutes within any 10-minute segment [10]. In addition, the patient presented with uterine tachysystole, which is defined as more than five contractions in 10 minutes, averaging over a 30-minute window [11]. When preparing a differential for this case, several factors must be investigated. However, because of the patient’s presentation, a uteroplacental cause was at the top of the list.

Uterine tachysystole itself can be a cause of fetal bradycardia, and there are several causes. Firstly, medications such as oxytocin, prostaglandins, and misoprostol can all induce labor and encourage uterine tachysystole [12]. Our patient did not use any of these medications as she was in uterine tachysystole upon arrival. During the initial assessment, the patient was on her hands and knees. The patient was instructed to move into different positions, such as the left lateral position, to help optimize blood flow to the placenta and the fetus, with the hopes that it would help mitigate fetal distress [13]. Furthermore, maternal conditions such as hypertension, preeclampsia, and uterine fibroids can all contribute to possible risk factors for uterine tachysystole. 

In addition to uterine tachysystole, placental insufficiency due to hypertensive disorders, preeclampsia, or intrauterine growth restriction (IUGR) can all cause fetal bradycardia [14]. As mentioned, the patient presented with an initial blood pressure reading of 130/85 mmHg, which does not technically meet the greater than 140/90 mmHg benchmark. However, given the patient’s history of preeclampsia and the relatively close numbers, a preeclampsia-related cause was highly likely. In fact, the patient may have been hypotensive because she was already actively bleeding from the abruption.

Finally, a placental abruption, which is often preceded by preeclampsia, was high on the differential list due to a variety of factors. Firstly, abruption is a significant risk factor for fetal bradycardia due to its disruption of fetal oxygen supply [2]. Also, the patient’s presentation of uterine tachysystole, sudden onset of abdominal pain, and a failure of fetal bradycardia resuscitation efforts points toward a more emergent cause.

Initial pertinent laboratory results showed mild leukocytosis and microcytic anemia, with a hemoglobin of 10.8. Lactate dehydrogenase (LDH) was mildly elevated. Urinalysis was positive for the presence of protein, glucose, white blood cells, red blood cells, and bacteria. Of particular concern, the protein concentration was markedly elevated at 628 mg/dL (reference: 0-20 mg/dL), and the protein-to-creatinine ratio was significantly increased at 19.50 (reference: 0.00-0.19). Many of the laboratory findings were not provided until after the emergency cesarean section. A summary of laboratory findings before and after cesarean section is presented in Table 1.

**Table 1:** Initial pertinent lab findings before and after the patient's cesarean section. WBC: white blood cell count; LDH: lactate dehydrogenase; PT: prothrombin time; PTT: partial thromboplastin time; FEU: fibrinogen equivalent units

Laboratory test	Patient value	Reference range	Units
White blood cell count (WBC)	11.9	4.0-11.0	×10³/µL
Hemoglobin (preoperative)	10.8	12.0-16.0	g/dL
Hemoglobin (postoperative)	5.5	12.0-16.0	g/dL
Lactate dehydrogenase (LDH)	428	110-210	U/L
Urinalysis-protein	628	0-20	mg/dL
Protein-to-creatinine ratio	19.50	<0.20	unitless (ratio)
Prothrombin time (PT)	44.1	11.0-13.5	seconds
Partial thromboplastin time (PTT)	92.5	25.0-35.0	seconds
D-dimer	>20.0	<0.5	µg/mL FEU
Fibrinogen	<60	200-400	mg/dL
Platelet count	129	150-450	×10³/µL
Lactate (postoperatively)	12.0	0.5-2.2	mmol/L

Final diagnosis and intervention

After an emergency cesarean section, the postoperative findings included the delivery of a live, viable infant demonstrating fetal growth restriction and the presence of a clot in the uterus after placental delivery, which echoed initial suspicion of placental abruption. Due to the significantly increased protein-to-creatinine ratio, history of preeclampsia, and sudden onset of symptoms, it was evident that this was a case of “hidden” preeclampsia that led to a placental abruption.

After about two hours post-surgery, a rapid response was called on the patient due to hypotension, with systolic blood pressure in the 70s mmHg and vaginal bleeding with concern for intraabdominal bleeding. Laboratory results revealed severe anemia with a hemoglobin level of 5.5 g/dL, elevated lactate of 12 mmol/L, prolonged INR of 3.3, and cryoprecipitate levels below 60 mg/dL. Initial management included intravenous fluid boluses, transfusion of two units of packed red blood cells and one unit of fresh frozen plasma, and arterial line placement for hemodynamic monitoring. She was then admitted to the critical care unit (CCU) for further management.

In the CCU, she was treated for circulatory shock with a brief phenylephrine infusion, which was successfully weaned off, and aggressive blood product resuscitation. Her preeclampsia was managed with a magnesium sulfate infusion and a nicardipine drip to avoid postpartum development of eclampsia [15]. For placental hemorrhage, she received uterotonics and utilized the JADA system (Jersey City, NJ: Organon). The JADA is a vacuum system used to control postpartum hemorrhage or uterine bleeding by encouraging the uterus to contract, thus compressing open blood vessels and slowing bleeding [16]. Her acute blood loss anemia and coagulopathy were addressed with an aggressive massive transfusion protocol, which is a 1:1:1 ratio of packed red blood cells, platelets, and fresh frozen plasma [16]. Additionally, for renal failure, metabolic acidosis, and electrolyte imbalances, she received aggressive hydration, bicarbonate therapy, and calcium replacement.

The American College of Obstetricians and Gynecologists defines postpartum hemorrhage as a cumulative blood loss greater than or equal to 1000 mL or blood loss accompanied by signs or symptoms of hypovolemia within 24 hours after the birth process (includes intrapartum loss), regardless of route of delivery [17]. Her estimated blood before and during surgery was 1378 mL, and by the time she reached the CCU, her estimated blood loss was over 2000 mL. In addition, laboratory results drawn during her initial CCU workup revealed significantly elevated levels of prothrombin time (44.1 seconds), partial thromboplastin time (92.5 seconds), and D-dimers (over 20 μg/mL). Her fibrinogen levels (less than 60 mg/dL) and platelet levels (129 K/μL) were significantly decreased. The pattern of increased PT, PTT, and D-dimers, decreased fibrinogen, and thrombocytopenia is a pattern characterized as disseminated intravascular coagulation (DIC) [18]. DIC is a systemic hypercoagulable condition that can cause both small and large blood clots, leading to impaired circulation and potential multiple organ dysfunction [19]. As clotting factors and platelets become depleted in a self-perpetuating cycle, excessive bleeding may occur, sometimes serving as the initial sign of DIC [20]. This condition usually arises as an acute complication in patients with severe, life-threatening illnesses, including placental abruption [20]. Thus, it was evident that our patient’s case of placental abruption led to an unfortunate and serious case of DIC after her cesarean section.

Follow-up and outcomes

The patient used the JADA system for around 24 hours. After removal, there was no evidence of bleeding. The patient stayed in the hospital for five days to improve blood levels and maintain close monitoring. By the time of discharge, she was tolerating a regular diet, ambulating, openly communicative, and had stable labs.

The patient was discharged home with detailed post-care instructions. She was advised to call if she developed a fever, signs of infection, heavy bleeding, persistent nausea or vomiting, severe or worsening pain, or any other concerning symptoms. Education and discharge instructions were reviewed including activity restrictions such as no lifting over 20 pounds and no vaginal insertion for six weeks. Her understanding was assessed and confirmed.

The patient followed up after one week and reported no significant problems. She was breastfeeding well and denied fevers, chills, nausea, emesis, and any redness or drainage from her incision site. Upon discharge, she was prescribed 325 mg of ferrous sulfate three times a day, 600 mg of ibuprofen every 6 hours/PRN, 30 mg of nifedipine one time a day, and 5 mg of oxycodone by mouth every 4 hours/PRN. She was instructed to stop taking her aspirin. The patient was tolerating the medication regimen and reported that her recovery was progressing well.

## Discussion

This case highlights a rare presentation of placental abruption secondary to undiagnosed or "hidden" preeclampsia, resulting in severe maternal and fetal complications, including disseminated intravascular coagulation (DIC). Placental abruption is a serious obstetric emergency with significant maternal and fetal morbidity, often associated with preeclampsia, hypertension, trauma, and a history of prior abruption [1,4]. While preeclampsia is a well-established risk factor for placental abruption, its atypical or subclinical presentations can delay recognition and management, increasing the risk of severe outcomes [6,7].

The patient in this case had a history of preeclampsia and was on prophylactic aspirin, following standard guidelines to reduce the risk of recurrence [8]. However, despite an initial blood pressure reading of 130/85 mmHg, she lacked overt hypertension at presentation, underscoring the challenge of diagnosing preeclampsia in cases where the classical signs are not evident. The markedly elevated protein-to-creatinine ratio (19.50) and significant proteinuria (628 mg/dL) postoperatively confirmed a diagnosis of preeclampsia that had not been clinically apparent prior to delivery. This aligns with literature suggesting that some cases of preeclampsia may manifest with minimal or atypical symptoms, leading to diagnostic delays and increased risk for complications such as placental abruption, eclampsia, and HELLP syndrome [7].

A crucial aspect of this case was the presentation of fetal bradycardia and uterine tachysystole, prompting emergent cesarean delivery. Uterine tachysystole has been linked to fetal distress, particularly in cases of placental insufficiency or abruption [12,13]. In this case, despite extensive resuscitative efforts, the persistently low fetal heart rate further supported an underlying placental pathology. The presence of a clot in the uterus upon placental delivery confirmed the suspected abruption, consistent with literature emphasizing fetal distress as a key indicator of placental abruption [2].

Following delivery, the patient experienced significant postpartum hemorrhage (PPH) exceeding 2000 mL, progressing to DIC. DIC is a well-known complication of severe placental abruption. The patient exhibited hallmark laboratory findings of DIC, including prolonged PT, PTT, elevated D-dimers, and thrombocytopenia. Rapid initiation of a massive transfusion protocol and uterotonic agents successfully stabilized the patient, consistent with best-practice management strategies for DIC and postpartum hemorrhage [18,19].

## Conclusions

This case reinforces several key takeaways. First, it underscores the importance of recognizing atypical presentations of preeclampsia, which may initially lack significant hypertension but present with proteinuria and end-organ dysfunction. Second, it highlights the diagnostic difficulty of placental abruption, particularly in cases with minimal or absent vaginal bleeding. Third, it demonstrates the critical need for prompt obstetric intervention in cases of fetal distress, as placental abruption can rapidly progress to life-threatening complications for both mother and fetus. Lastly, it emphasizes the role of aggressive resuscitation and multidisciplinary management in addressing severe postpartum hemorrhage and DIC. Ultimately, this study serves as a reminder for clinicians to maintain a high index of suspicion for preeclampsia and placental abruption, even in the absence of classic symptoms, to improve maternal and fetal outcomes through early detection and timely intervention.
